# Vision-Based Pedestrian’s Crossing Risky Behavior Extraction and Analysis for Intelligent Mobility Safety System

**DOI:** 10.3390/s22093451

**Published:** 2022-04-30

**Authors:** Byeongjoon Noh, Hansaem Park, Sungju Lee, Seung-Hee Nam

**Affiliations:** 1 Applied Science Research Institute, Korea Advanced Institute of Science and Technology, 291 Daehak-ro, Yuseung-gu, Daejeon 34141, Korea; powernoh@kaist.ac.kr; 2 Department of Civil and Environmental Engineering, Advanced Institute of Science and Technology, 291 Daehak-ro, Yuseung-gu, Daejeon 34141, Korea; saem@kaist.ac.kr; 3 Department of Software, Sangmyung University, Cheonan 31066, Korea; 4 Center for Accelerator Research, Korea University, Sejong 30019, Korea

**Keywords:** crossing behavior analysis, pedestrian safety, potential collision risks, computer vision

## Abstract

Crosswalks present a major threat to pedestrians, but we lack dense behavioral data to investigate the risks they face. One of the breakthroughs is to analyze potential risky behaviors of the road users (e.g., near-miss collision), which can provide clues to take actions such as deployment of additional safety infrastructures. In order to capture these subtle potential risky situations and behaviors, the use of vision sensors makes it easier to study and analyze potential traffic risks. In this study, we introduce a new approach to obtain the potential risky behaviors of vehicles and pedestrians from CCTV cameras deployed on the roads. This study has three novel contributions: (1) recasting CCTV cameras for surveillance to contribute to the study of the crossing environment; (2) creating one sequential process from partitioning video to extracting their behavioral features; and (3) analyzing the extracted behavioral features and clarifying the interactive moving patterns by the crossing environment. These kinds of data are the foundation for understanding road users’ risky behaviors, and further support decision makers for their efficient decisions in improving and making a safer road environment. We validate the feasibility of this model by applying it to video footage collected from crosswalks in various conditions in Osan City, Republic of Korea.

## 1. Introduction

Despite advances in vehicle safety technologies, road traffic accidents globally still pose a severe threat to human lives and have become a leading cause of premature deaths [1]. Every year, approximately 1.2 million people are killed and 50 million injured in traffic accidents [2,3]. Among the various types of traffic accidents, in the case of a vehicle–pedestrian collision, pedestrians are especially exposed to various hazards, such as drivers failing to yield to them at crosswalks [2]. According to international institutes, such as British Transport and Road Research Laboratory and World Health Organization (WHO), crossing roads at unsignalized crosswalks is as dangerous for pedestrians as crossing roads without crosswalks or traffic signals [4].

There are a variety of ways to prevent vehicle–pedestrian collisions, such as suppressing dangerous or illegal behaviors of road users (mainly vehicles and pedestrians) by deploying speed cameras and fences, and operating 24 h CCTV surveillance centers in administrative districts. In addition, some studies have analyzed actual collisions and their factors [5,6], and suggested countermeasures. However, such approaches have used historical accident data or metadata to improve the safety of road environments post facto. Therefore, it is necessary to devise strategies to proactively respond to such collisions.

One of the breakthroughs is to analyze potential risky behaviors of road users (e.g., near-miss collisions), which can provide clues to take action such as deployment of additional speed cameras, speed bumps, and other traffic calming measures [2,7,8,9,10,11]. The investigation of the risky behaviors, which are heavily influenced by road users’ emotions, will help in improving road safety. In order to capture these subtle potential risky situations and behaviors, vision sensors are employed, such as closed-circuit televisions (CCTVs) on the roads. The use of vision sensors is supposed to make it easier to study potential traffic risks over long periods of time, and allows analyses such as evaluating the behavioral factors that pose a threat to pedestrians at crosswalks based on vehicle–pedestrian interactions [7,8,12,13], and supporting decisions based on their subtle interactions [9,10]. 

In general, one of the most important steps in vision-based traffic safety and surveillance systems is to obtain the behavioral features of vehicles and pedestrians from the video footage. However, the vision-based approach has a critical problem. Since most CCTVs are already deployed with oblique views of the road, it is difficult to obtain precise coordinates and behavioral features such as objects’ speeds and positions. Thus, many studies have used manual inspection to reliably extract these features from video footage. This requires more cost and time when extended to the urban scale, so we should address these challenges when seeking to analyze pedestrian safety across many sites in the city.

This study introduces a new approach to obtain the potential risky behaviors of vehicles and pedestrians from CCTV cameras deployed on the roads. This research begins with the question: can the subtle behaviors and intentions of vehicles and pedestrians be understood from video footage. Thus, the objectives of this study are: (1) to process the video data as one sequence from motioned-scene partitioning to object tracking; (2) to extract automatically the behavioral features of vehicles and pedestrians affecting the likelihood of potential collision risks between them in crosswalks; and (3) to analyze behavioral features and relationships among them by camera location. This study follows earlier experiments using sample footage [14], but improves on the methods and expands to a much larger dataset covering nine cameras over two weeks. The remainder of this paper consists of five sections described as follows:

1. Literature Review: Reviewing the related works for vehicle–pedestrian’s risky behavior analysis and vision-based traffic safety system.
2. Data Arrangement: Description of test spots and overview of the video dataset and preprocessing methods.
3. Potential Collision Risky Behavior Extraction: Description of methods for object’s behavioral extraction.
4. Performance Evaluation: Validation of preprocessing results.
5. Analysis of Potential Collision Risky Behaviors: Analysis of the objects’ behavioral features by spots, and discussion of results and limitations.
6. Conclusion: Summary of our study and future research directions.

To the best of our knowledge, it the is first attempt to understand and analyze the subtle behaviors of road users, from video footage. This study has three novel contributions: (1) recasting CCTV cameras for surveillance to contribute to the study of pedestrian environments; (2) creating one sequential process from detecting objects to extracting their behavioral features; and (3) analyzing the extracted behavioral features and clarifying the interactive moving patterns by crossing environment. Consequently, the proposed method can handle the video stream in order to obtain objects’ behaviors in multiple spots. These kinds of data are the foundation for understanding road users’ risky behaviors, and further support decision makers for their efficient decisions in improving and making a safer road environment. We validate the feasibility of this model by applying it to video footage collected from crosswalks in various conditions in Osan City, Republic of Korea.

## 2. Materials and Methods

To achieve the purposes of this study requires both handling the vision-based road traffic data and analyzing potential collision risky behaviors, especially for vehicle–pedestrians. In this section, we briefly introduce the literature for vehicle–pedestrian risky behavior analysis, and further vision-based traffic safety systems.

### 2.1. Vehicle–Pedestrian’s Risky Behavior Analysis

In order to make up for actual traffic accidents’ shortcomings, some studies aim to analyze potential collision risks by using behavior and characteristics of road users [14,15,16] and environmental factors [12,17]. For example, the authors in [14] analyzed a variety of factors contributing to pedestrian safety such as pedestrian’s walking phase, speed, and gap acceptance by countries. These results can give guidance to decision makers and administrators with useful and powerful information supporting to improve and make a safe traffic environment. Similarly, the authors in [16] investigated the pedestrian’s crossing speed, delays, and gap perceptions at signalized intersections. They applied an analysis of variance (ANOVA) method to reveal the factors affecting the pedestrian walking speed and safety margin. Furthermore, the authors in [15] investigated the age effect of pedestrian road-crossing behaviors and described how age affects street-crossing decisions with vehicle speed, time gap, and time of day, together.

In terms of environmental factor analysis, the authors in [12] provided an informative tool for evaluating the collision risk between vehicles and pedestrians for improving pedestrian safety in urban environments. In order to evaluate the collision risks, they used features such as pedestrian counts and automobile traffic flow, and identified a safety in numbers effect. The authors in [18] studied the relationships between pedestrian risks and the built environment. They figured out that pedestrian road traffic injuries depend on the design of the roadway and land uses.

Meanwhile, the authors in [17] analyzed the vehicle–pedestrian near-crash identification using the trajectories of vehicles and pedestrians extracted from roadside LiDAR data. The study focused on identifying vehicle–pedestrian near-crash, especially considering the increased risk of vehicle–pedestrian conflicts. To identify the near-crash between vehicle and pedestrian, three parameters—Time Difference to the Point of Intersection (TDPI), Distance between Stop Position and Pedestrian (DSPP), and vehicle–pedestrian speed–distance profile were developed used in the research. However, the performance of near-crash identification using the three developed parameters was not stable. To increase the accuracy, the authors in [19] proposed an improved vehicle–pedestrian near-crash identification method with three indicators: Post-Encroachment Time (PET), the Proportion of the Stopping Distance (PSD), and the Crash Potential Index (CPI). The case studies show that the proposed method can evaluate pedestrian safety without waiting for historical crash records.

In this study, we also focus on analyzing potential collision risky behaviors between vehicles and pedestrians such as near-miss collisions, not actual collisions. However, unlike the existing studies, we use vision-based data sources, and further extract the various behavioral features for analysis.

### 2.2. Vision-Based Traffic Safety System

There have been many efforts to build a vision-based transportation system, especially focusing on safety. For example, the authors in [11] proposed an onboard monocular vision-based framework to automate the detection of the near-miss event data. The advantages of the onboard monocular camera are the large coverage area and numerous data sources. In the research, time-to-collision (TTC) and distance-to-safety (DTS) are used in near-miss detection. Similarly, the authors in [20] focused on near-miss incidents by using the driving records installed in passenger vehicles. Specifically, TTC was calculated to analyze the potential risk between pedestrians and vehicles based on the video frames captured by the drivers’ records. The results indicate that the average TTC is shorter when the pedestrians are not using the pedestrian crossing and emerged from behind obstructions. The authors in [21] proposed a new analytical system for potential pedestrian risk scenes based on video footage obtained by road security cameras already deployed at unsignalized crosswalks. Similarly, the authors in [22] proposed a new framework for a vision sensor-based intersection pedestrian collision warning system (IPCWS) that gives a collision warning to drivers approaching an intersection by predicting the pedestrian’s crossing intention based on various machine learning algorithms. Furthermore, the authors in [22] considered the 3D pose estimation factor in real-time to clarify the pedestrian’s intention of crossing. The authors in [23] also investigated vehicle–pedestrian behaviors by using the vision-based data, and they focused on analyzing instant behaviors of them in the video stream.

In this study, we also focus on extracting objects’ behavioral features, especially risky behaviors, from video footage and analyzing them. In fact, there are many kinds of measurements of risky behaviors, especially surrogate measurements, such as speed TTC and DTS, as well as speeds and distances of vehicle and pedestrian. In our experiment, we handle the overall behavioral features such as speed, distance, and pedestrian safety margin (PSM) with a focus on extracting them automatically, and then evaluating the performance of the extracted features from video.

## 3. Data Arrangement

In this section, we describe the video dataset used in our experiment and how to extract the behavioral features of vehicles and pedestrians that might affect the likelihood of potential collision risks between them in a visual environment. First, we process the given input video stream from CCTV cameras, called preprocessing, consisting of three steps: (1) motioned-scene partitioning; (2) object detection in overhead view; and (3) object tracking. As the outputs, we can obtain the objects’ trajectories, and, then, the objects’ behavioral features are extracted from these trajectories.

### 3.1. Data Sources

In our experiments, we use video data from CCTV cameras deployed on nine roads in Osan City, Republic of Korea. The information for each spot is arranged in Table 1, including road characteristics and recording metadata. These cameras are deployed over crosswalks, and are intended to record and deter instances of street crime. Some are deployed in school zones, which are certain roads near facilities for children under age 13, e.g., elementary schools, daycare centers, and tutoring academies. Penalties for breaking traffic rules or causing accidents in these areas are highly severe, such as fines of up to KRW 3000 million or life imprisonment, in order to suppress risky behavior [24]. 

All video frames were processed locally on a computer server we deployed in the Osan Smart City Integrated Operations Center, and we only obtained the processed trajectory data after removing the original video data. This was to protect the privacy of anyone appearing in the footage. Future systems could employ internet-connected cameras that process images on-device in real time, and transmit only trajectory information back to servers.

**Figure 1:**
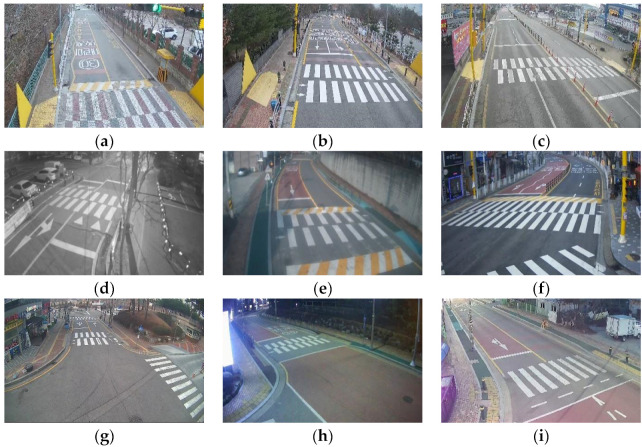
Actual CCTV views in (**a**) Spot A; (**b**) Spot B; (**c**) Spot C; (**d**) Spot D; (**e**) Spot E; (**f**) Spot F; (**g**) Spot G; (**h**) Spot H; and (**i**) Spot I.

Figure 1a–i show the CCTV views being actually recorded in spots A to I, respectively. Since these spots have a high “floating population” during commuting hours, due to their proximity to schools and residential complexes, we used video recorded on weekdays from 9 to 28 January 2020, from 8 a.m. to 10 a.m., and from 6 p.m. to 8 p.m.

### 3.2. Preprocessing

#### 3.2.1. Motioned-Scene Partitioning

**Figure 2:**
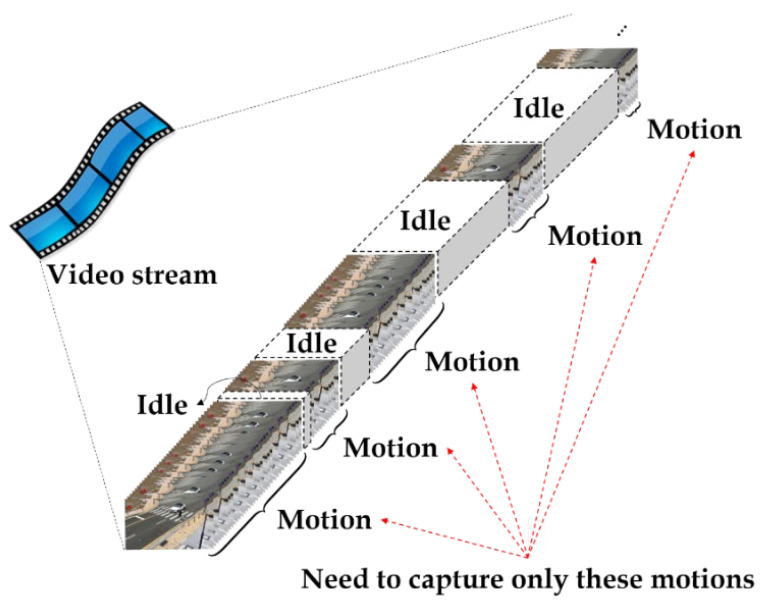
Composition of the actual video stream.

As a first step of preprocessing, we partition the video stream into only video clips with moving vehicle or pedestrian activities, regarded as “motioned-scene.” The goal of this step is to make efficient processing of video footage. In general, there are occasionally some motioned-scenes (see Figure 2), but CCTVs on the road constantly record for 24 h, so most frames are idle states. Thus, it is necessary to decide whether to process the input frame or not. Thus, it requires a method with a simple and low computational complexity to handle the video footage.

For this, we apply a frame difference method, a widely used approach for detecting moving objects from the fixed cameras [25,26]. This method simply calculates the pixel-based difference between two frames, as an image obtained at the time t, denoted by *I(t)*, and the background image denoted by *B*:

(1) $$P\left[F\left(t\right)\right]=P\left[I\left(t\right)\right]-P\left[B\right]$$

where pixel value in *I(t)* is denoted by *P*[*I*(*t*)], and *P*[*B*] means the corresponding pixels at the same position on the background frame. As a result, we can observe the intensity of the pixel positions that have changed in the two frames, and then detect the “motion” by comparing it with the threshold as follows:

(2) $$\left|P\left[I\left(t\right)\right]-P\left[I\left(t+1\right)\right]\right|>Threshold\;\;$$

**Figure 3:**
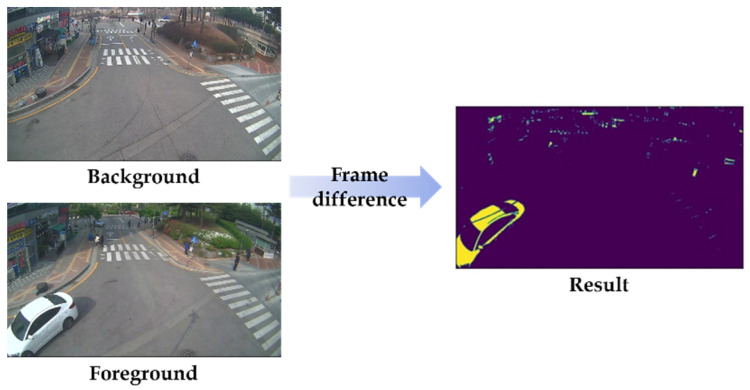
Example of frame difference.

The example of frame difference is illustrated in Figure 3. In practice, the frame difference method is applied to all frames, and if a motion is recognized in the given two consecutive frames, the following algorithms work.

#### 3.2.2. Object Detection in Overhead View

Next, objects in motioned-scene are detected by using deep learning-based object detection models. We used a mask R-CNN (Regional Convolutional Neural Network) model, an extension of faster R-CNN, which was a pre-trained model with ResNet-101-FPN by Microsoft common objects in context (MS COCO) image dataset [27]. In our experiment, we use the Detectron 2 platform, as implemented by Facebook AI Research (FAIR) [28]. Since the accuracy was close to perfect for these objects in our video footage, this pre-trained model did not need to be trained further for our purposes. As the output of object detection, we can obtain the bounding-box information with four x-y pixel coordinates for each object.

Typically, road-deployed CCTV cameras record from oblique views, so it is difficult to precisely extract their behavioral features such as speeds and positions. To solve this, we recognize the “ground tip” points of the vehicle and pedestrian, which are situated directly underneath the front bumper and on the ground between the feet, respectively. The ground tip point of the vehicle is captured by using the object mask matrix, as output from the mask R-CNN model, and the central axis line of the vehicle lane, and one of the pedestrians is regarded as the midpoint from its tiptoe points within the mask. Then, the perspectives of the obtained ground tip points are transformed into the top view. More detailed procedures for this transformation are explained in our previous studies [23,29].

#### 3.2.3. Object Tracking

**Figure 4:**
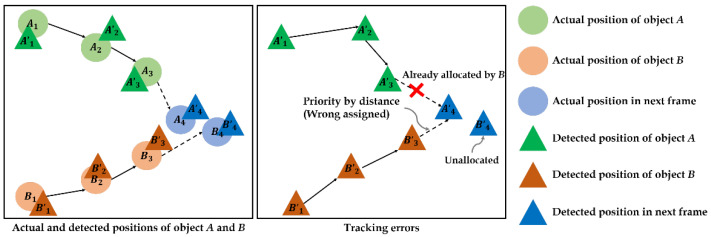
Example of the actual movements of two objects (**left**); and tracking errors (**right**).

Lastly, we identify each object in a consecutive frame by using an object tracking algorithm. In our experiment, we improved an existing object tracking algorithm from our previous works using a centroid track with threshold and minimum distance methods [30]. This previous algorithm accounts for distance when postulating the location that an object can move to in the next frame, prioritizing the closest object rather than the most likely one. However, this makes some errors; other objects are regarded as having disappeared out of frame if their distance to the remaining positions is greater than the threshold. Furthermore, in the vision-based object handling process, there is noise at the positions of the detected objects, so it is difficult for the previous object tracking algorithm to cope with this issue, as illustrated in Figure 4. Assume that there are two objects, *A* and *B*, in multiple consecutive frames, and the trajectories of *A* and *B* from frames 1–3 are, so far, already connected, and are trying to correctly assign $A_{4}$ and $B_{4}$. The circular positions mean the actual positions of each object, and the triangular ones mean the detected positions in the video by using an object detection model. In practice, the contact points of the object have a noise due to either the object detection model or contact point recognition process, so there is a slight difference between the actual object’s position and the detected position, affecting the performance of the object tracking. Thus, it is necessary to improve the accuracy of the tracking algorithm by adjusting this noise.

To address these errors, we applied a modified Kalman filter method to more accurately track objects from frame to frame. Much research has been conducted on object tracking and indexing in various fields of computer science and transportation [30,31,32]. In particular, Kalman filters have been used in a wide range of engineering applications such as computer vision and robotics. They can efficiently calculate the state estimation process [33] and can be applied to estimate the unknown current or future states of the objects in the video [34]. A Kalman filter calculates the next position of an object by repeatedly performing two steps: (1) state prediction, and (2) measurement update. In the state prediction step, the current object’s parameter values are predicted using previous values such as positions and speeds. In the measurement update step, the parameter values of the current object are updated by using the prior predicted values and information obtained about the current object’s position.

**Figure 5:**
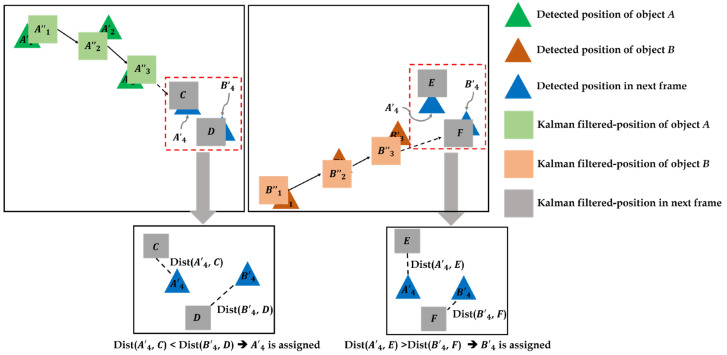
Process of object tracking and indexing algorithm for object *A* (**left**) and object *B* (**right**).

The tracking and indexing algorithm used in this study consists of two parts: (1) estimating the candidate points based on smoothing; and (2) assigning objects in the next frame by calculating and comparing distances. First, we smooth the existing trajectory points using a Kalman filter to make positions and speeds more consistent. Then, we predict the next location of the trajectory, and calculate all distances between this and the candidate locations in the next frame, choosing the closest match. Unlike the previous object tracking method (no Kalman filter), the modified Kalman filter-based object tracking method has a smoothing step, so it can adjust the noisy positions of objects. As represented in Figure 5, we smooth the trajectories through frames 1–3, and predict the object’s position in frame 4. The smoothed points are represented as rectangles denoted with doubled-apostrophes such as ${A^{\prime \prime }}_{1}$ ${A^{\prime \prime }}_{2}$, and ${B^{\prime \prime }}_{3}$ and the estimated target objects are denoted *C*, *D*, *E*, and *F*. Next, we calculate the distances between the origin target objects and the estimated target objects, denoted $Dist\left(origin\;target\;object,\;estimated\;target\;object\right)$. Finally, the target object with the smallest distance from its prediction is assigned to the trajectory, and this process is repeated until the last frame in scene.

As a result, we extracted about 50,000 scenes from the entire video dataset, and used 45,890 scenes involving traffic-related objects as seen in Table 2. Each scene spanned approximately 38 frames, or 1.38 s. The majority of scenes captured only passing cars, while “interactive scenes” involved both vehicles and pedestrians in the scene at the same time. Finally, we obtained the scenes with trajectories of vehicles and pedestrians in video footage, and preparations for extracting their behavioral features are completed.

## 4. Potential Collision Risky Behavior Extraction

In this section, we describe which behavioral features were extracted and how to automate these processes. In fact, there are many kinds of indicators to measure potential collision risks, but practically it is difficult to handle all of them. Thus, in our experiment, we extracted about 10 features among plenty of such features that could relate to potential collision risky behaviors as seen in Table 3, and the extracting methods are described below in detail.

**Vehicle and pedestrian speeds**: In general, object speed is a basic measurement that can signal potential risky situations. Car speed is a significant risk factor for pedestrian fatalities, and has a close relationship with crash severity in vehicle-to-pedestrian collisions [35,36]. Speed limits in our all testbeds were 30 km/h. A large number of detected vehicles traveling over the limit at any point, especially in school zones, contributes to high potential risk at that location. Meanwhile, pedestrian speed alone is not a direct indicator of such risks, but we may find important correlations and interactions with other features such as vehicle speed and vehicle–pedestrian distance.

Object speed can be obtained from an assembled trajectory by the dividing distance between its position in two consecutive frames by the time interval. In this case, the pixel distance between point i in $j^{\mathrm{th}}$ and ${\left(j+1\right)}^{\mathrm{th}}$ frames in x-y plane, $D_{pixel}\left(point_{i}^{j},\;point_{i}^{\left(j+1\right)}\right)$, is computed by the Euclidean distance method, and converted into real-world distance units such as meters. We infer the pixel-per-meter constant, denoted as P, by dividing the pixel length of the crosswalk ($l_{pixel}$) by the actual length of it ($l_{world}$); we measured the actual lengths of crosswalks in field visits. For example, if the length of a crosswalk is 15 m, and the pixel length is 960 pixels, 1 m is about 46 pixels (=960/15).

Meanwhile, the frame intervals between trajectory points must be converted to real-world seconds. The time conversion constant (*F*) is computed by dividing the skipped frames by FPS. For example, if the video is recorded at 11 FPS, and we sampled every fifth frame, the time interval *F* is equal to 5/11. Finally, $i^{th}$ object’s speed in $j^{th}$ and ${\left(j+1\right)}^{th}$ frames can be calculated as follows:

(3) $$Speed_{i}^{j,\;\left(j+1\right)}=\frac{D_{pixel}\left(point_{i}^{j},\;\;point_{i}^{\left(j+1\right)}\right)\;}{F*P}\;\;\left(m/s\right)$$

Finally, we convert these measurements into km/h, and apply them to all frames in the scene to obtain the instantaneous object speeds in each frame. As a result, the speed list of object *i* in scene *k* consisting of *j* frames is represented as:

(4) $$speedList_{k,\;i}=\left[\;speed_{i}^{1,2},\;speed_{i}^{2,3},\;speed_{i}^{3,4}\ldots \;speed_{i}^{\left(j-1\right),j}\right]$$

**Figure 6:**
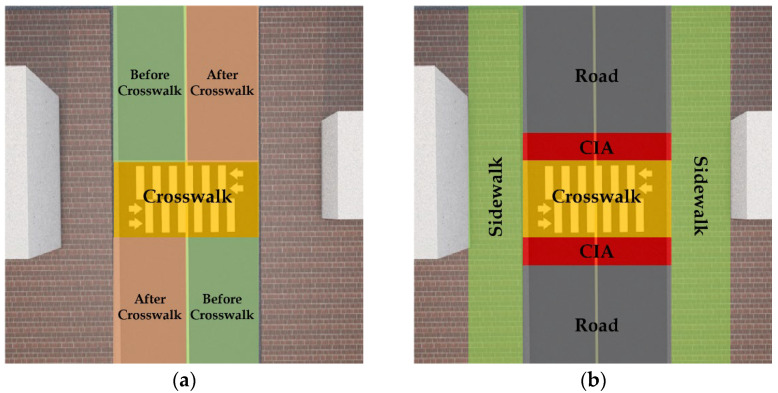
Categories of positions for: (**a**) vehicle, and (**b**) pedestrian.

**Vehicles and pedestrian’s positions**: The objects’ positions on the road are also important to investigate the potential traffic risks. A pedestrian on the road, even when cars are moving at a slow speed, may be more at risk than a pedestrian on the sidewalk when cars are moving at high speed. In this study, the vehicle’s position is categorized into three areas: “before crosswalk”, “on crosswalk”, and “after crosswalk”, and the pedestrian’s position is categorized into four areas using their coordinates: “sidewalk”, “crosswalk”, “crosswalk-influenced area (CIA)”, and “road”. CIA refers to the road area adjacent to the crosswalk, where pedestrians often enter while crossing the road [37,38,39]. The detailed areas are illustrated in Figure 6a,b, respectively. In this study, we encompassed CIA with a buffer of ~3 m on either side of the crosswalk.

**Figure 7:**
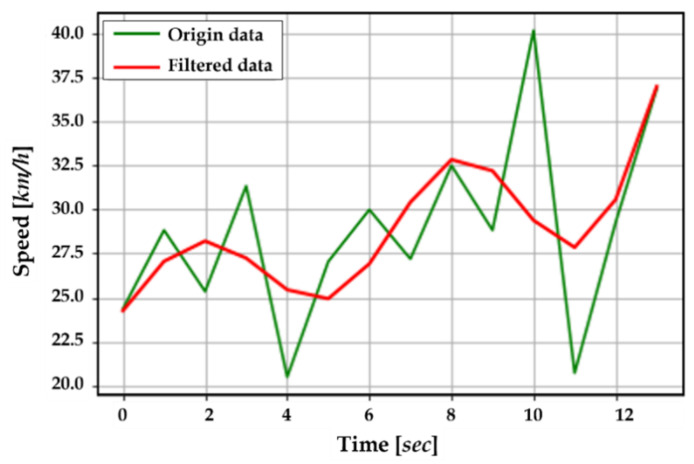
The origin speeds (green line) and the filtered data (red line).

**Vehicle acceleration**: Vehicle accelerations and their changes during the scene are important factors to consider; if many vehicles maintain their speed or accelerate while approaching the crosswalk, this increases the risk to pedestrians. Ideally, we would expect to see cars decelerate near crosswalks, especially when pedestrians are present. In our experiment, we categorized vehicle accelerations as “acc”, “dec”, and “nc” by considering only speed changes. First, we smooth the speed sequence (see Figure 7) using a low-pass filter method, commonly used to reduce the rapid fluctuation of the signal that may result from the imprecision of object positioning from the image processing algorithm [40,41]. This results in the filtered speed list, $F\left(velList_{k,\;i}\right)$, with the filtered values, *f*$\left(velList_{i}^{j,\;\left(j+1\right)}\right)$, where the subscripts k and i are scene number and object number in this scene, respectively.

Next, we calculated slope changes in the graph (means vehicle acceleration in the time–speed graph) from when the vehicle enters the scene to when it reaches the crosswalk. We classified these as a sequence of acceleration states, with positive slopes yielding “acceleration”, negative as “deceleration”, and close to zero as “no change”. This procedure can be written in mathematic equations as follows:

(5) $$Acc_{i}^{j}=\left\{\begin{array}{cc} ”acc”, & f\left(speed_{i}^{\left(j+1\right),\;\left(j+2\right)}\right)-f\left(speed_{i}^{j,\;\left(j+1\right)}\right)>\varepsilon \\ ”dec”, & f\left(speed_{i}^{\left(j+1\right),\;\left(j+2\right)}\right)-f\left(speed_{i}^{j,\;\left(j+1\right)}\right)<\varepsilon \\ ”nc”, & otherwise \end{array}\right.$$

**Vehicle stop before crosswalk**: This feature indicates whether the vehicles came to a stop, before passing the crosswalk. Vehicles at these locations were required to stop once before passing the crosswalk, with or without pedestrians present. In practice, since the values of the extracted speeds have noise, we used the concept of “speed tolerance” to detect stops. The descriptions of speed tolerance will be elicited in the experiment part, and the details on speed tolerance are described in our previous study, [23].

**Crosswalk distance and vehicle–pedestrian distance**: Crosswalk distance list means the distance changes between vehicles and crosswalk by frame, while the vehicle–pedestrian distance list measures the sequence of distances between the vehicle and nearest-pedestrian by frame. Distances between vehicle *i* and pedestrian *p* are ordered by frame as follows:

(6) $$dist_{i,p\;}^{j}=\frac{D_{pixel}\left(vehicle_{i}^{j},\;\;pedestrian_{p}^{j}\right)}{P}\;\left(m\right)$$

(7) $$distChng_{k,i,p}=\left[\;dist_{i,p\;}^{1},\;\;\;dist_{i,p\;}^{2},\;\;\;dist_{i,p\;}^{3},\;\;\;\ldots ,\;dist_{i,p\;}^{j}\right]$$

where the subscripts *k* and *j* are scene number and the frame order, respectively.

**Figure 8:**
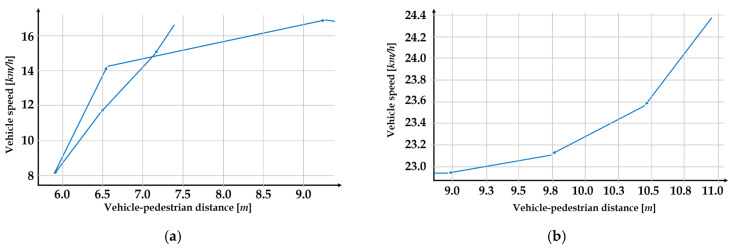
Examples of analyzing vehicle–pedestrian distance and other features; **(a)** slowing down and dramatically accelerated; and **(b)** normal slowing down when approaching to pedestrian.

These distance sequences alone are not factors for potential risk, but when compared with other features, we may identify dangerous situations. For example, Figure 8a,b show two scenes as vehicle speeds plotted against vehicle–pedestrian distances while the pedestrian was on the crosswalk. In these examples, assume that the vehicle speed is not considered if it does not exceed the speed limit, and only investigate its changes by vehicle–pedestrian distance. 

In Figure 8a, we can observe that as the vehicle approached the pedestrian, its speed decreased rapidly, then accelerated again immediately after the pedestrian passed. Although the vehicle slowed down when needed, it also accelerated rather rapidly even before the pedestrian had safely reached the sidewalk. In Figure 8b, the vehicle slows down as it approaches the pedestrian, and the speed is under the speed limit (almost 30 km/h). Now, we cannot determine which is more dangerous, but when considering only patterns of vehicle speeds, Figure 8a is a pattern of re-acceleration after deceleration, and Figure 8b is a pattern of continuous deceleration. These figures are just examples that have the possibility to identify dangerous situations using the shapes of these features with others together.

**Relative position change between vehicles and pedestrians**: This describes the positional relationship between vehicles and pedestrians. If a pedestrian is in front of the car, they are at greater risk than if they were behind the car. We determine the relative positions between them by comparing their contact points, along with the position and direction of the vehicles.

This alone is not an obvious signal for risk, but when analyzed together with other features such as vehicle speed and pedestrian position, we may find important correlations and interactions between them. For example, a pedestrian who is behind a vehicle and on the sidewalk is in a relatively safe position.

**Pedestrian safety margin (PSM)**: There are various ways to define the concepts of PSM [15,42,43,44]. In this study, we defined PSM as the time difference between when a pedestrian crossed the conflict point and when the next vehicle arrived at the same conflict point [42,45,46]. Suppose a pedestrian reaches a conflict point at time $T_{1}$, and the vehicle arrives at the same conflict point at time $T_{2}$, then the PSM is $T_{2}-T_{1}$. Smaller PSM values mean there is less margin for error to avoid a collision at the conflict point.

**Figure 9:**
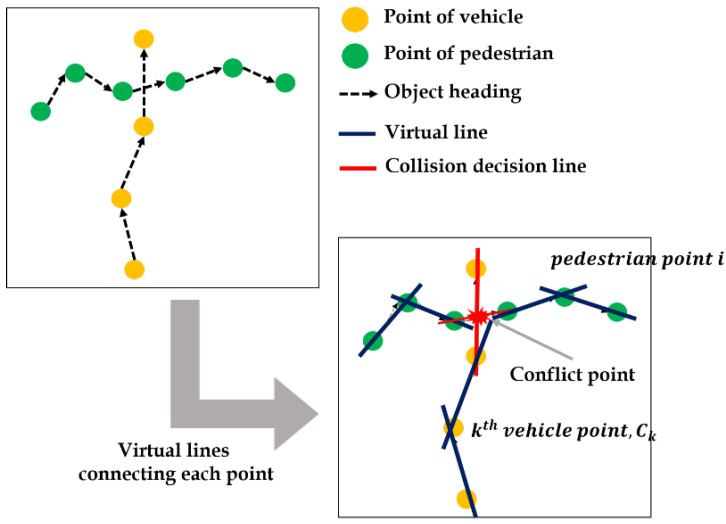
Expected conflict point in object trajectories.

Since the goal of this study is to extract these behavioral features automatically, it is important to infer the conflict point as seen in Figure 9. In this study, we applied virtual lines connecting the same objects between consecutive frames, and used the intermediate value theorem (IVT).

**Figure 10:**
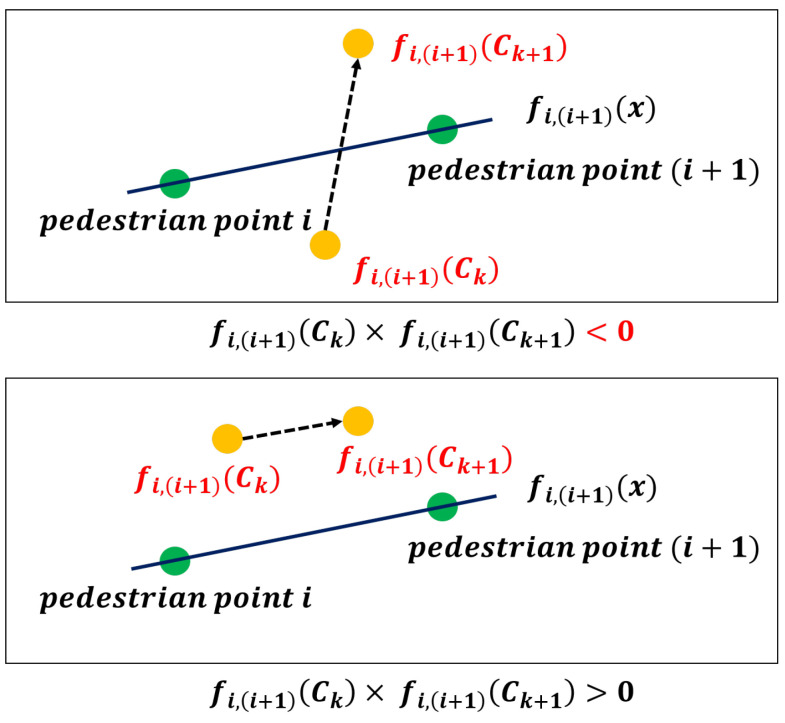
Process of finding conflict points by using IVT.

As represented as Figure 10, the process of PSM value extraction follows three steps: (1) drawing the virtual lines connecting the points of pedestrian in $i^{th}\;$ and ${\left(i+1\right)}^{th}$ frames, functionalized as linear function $f_{i,\left(i+1\right)}\left(x\right)$; (2) multiplying function values, $f_{i,\left(i+1\right)}\left(C_{k}\right)$ and $f_{i,\left(i+1\right)}\left(C_{k+1}\right)$ where $C_{k}$ and $C_{k+1}$ are vehicles points, respectively; and (3) iterating steps 1 and 2 for all points in trajectories until $f_{i,\left(i+1\right)}\left(C_{k}\right)\times f_{i,\left(i+1\right)}\left(C_{k+1}\right)$ is negative.

Applying IVT this way results in either a positive or negative value; if the result is positive, these points i and k are not in conflict. If it is negative, there is a conflict point between these points, and we can obtain the PSM values by calculating the difference between i and k, and adjusting the time unit from frames into seconds, as follows:

(8) $$find\;\;\;i,\;k\;\;\;\;\;s.\;\;t.\;\;\;\;f_{i,\left(i+1\right)}\left(C_{k}\right)\times f_{i,\left(i+1\right)}\left(C_{k+1}\right)<0$$

(9) $$PSM=\frac{\left(i-k\right)}{F}\;\;\left(sec\right)$$

## 5. Performance Evaluation

### 5.1. Experimental Design

Prior to potential collision risk analysis, we validate the results of preprocessing of vision-based data: (1) object tracking, and (2) behavior extraction.

**Figure 11:**
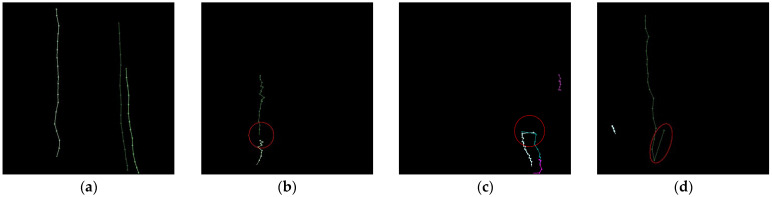
Trajectories of (**a**) the correctly tracked objects in scenes, and violating three criteria; (**b**) connectivity; (**c**) crossing; and (**d**) directivity.

First, in order to validate the object tracking algorithm, we defined success criteria, and manually counted all scenes with trajectories of objects that violated these criteria. Figure 11a shows trajectories for correctly tracked objects. As seen in these figures, the trajectories of objects should be continuous, and two or more objects should not cross each other. In addition, since this algorithm applied a threshold method, if there are unallocated objects within the threshold range, they could be traced incorrectly. Thus, we defined three criteria as follows:

- Connectivity: Are all of the objects connected in consecutive frames without breaks?
- Crossing: Are two or more objects, moving in parallel, traced separately without intertwining?
- Directivity: Do the objects follow their own paths without invading others’ trajectories? This phenomenon may occur more frequently when adjusting the threshold.

Figure 11b–d represent scenes that violate the above three criteria, respectively.

As a baseline, we compare the object tracking algorithm without the Kalman filter (previous method in our work) with the used one.

Next, we evaluate the behavior extraction method. Since the performance of the extracted behaviors, especially the object’s speed and acceleration, depends on the accuracy of the object’s coordinates calculated in the “object detection step” in preprocessing. It means that distance has some level of error, and speed and acceleration also have some level of error. Thus, we aim at obtaining the precise contact points, and then derive speed/acceleration errors. In fact, it is difficult to clarify a point that exactly represents the contact point of the vehicle or pedestrian in a mono-vision sensor, so we adopt a concept of “distance tolerance”, denoted by $\varepsilon_{dist}$, which tolerates some errors by assuming that if there are calculated contact points within the error boundary, these points are properly recognized.

In order to evaluate the accuracy of the contact points, we asked the recruited 12 testers to choose the pixel location for the actual contact points of the vehicle and pedestrian for top-view-converted 100 frames, respectively. Then, we measure the accuracy by various distance tolerance (10 cm, 20 cm, 35 cm, 50 cm, 60 cm, and 70 cm) by comparing the difference between the points derived from the proposed method and the points by testers.

### 5.2. Result

#### 5.2.1. Evaluation of Object Tracking Algorithm

The result of validation is shown in Table 4. We compared our tracking and indexing algorithm with our prior simple algorithm (see [29]). As a result, the overall accuracy is approximately 0.9, and the average accuracy is about three percent higher than that of the existing method. In particular, by using the Kalman filter, the accuracy of directivity increased about two percent.

#### 5.2.2. Evaluation of Behavior Extraction Method

Table 5 shows the average accuracy of contact point recognition in each spot by objects. As a result of the comparison, the average accuracies for both vehicle and pedestrian are more than about 0.89 when the distance tolerance is more than 50 cm. Although the distance tolerance with the best performance is 70 cm (accuracies are about 0.95 and 0.93 for vehicle and pedestrian, respectively), the distance tolerance of 50 cm is the best option when considering a speed tolerance, $\varepsilon_{v}$.

**Figure 12:**
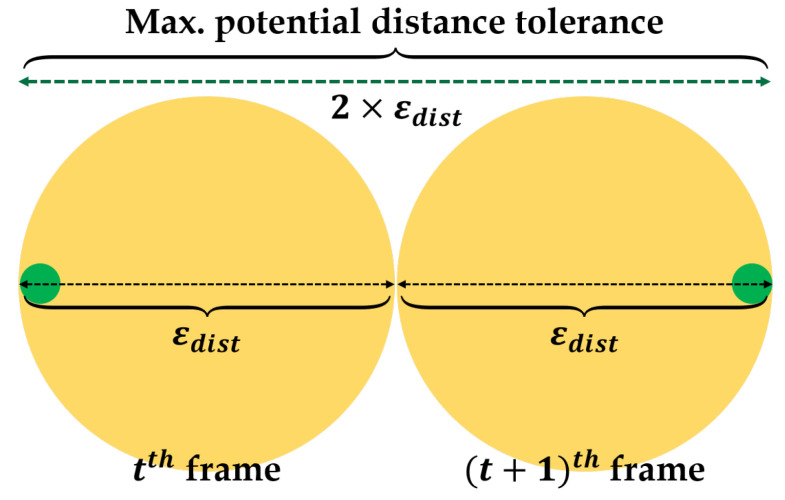
Speed tolerance based on maximum potential distance tolerance.

**Figure 13:**
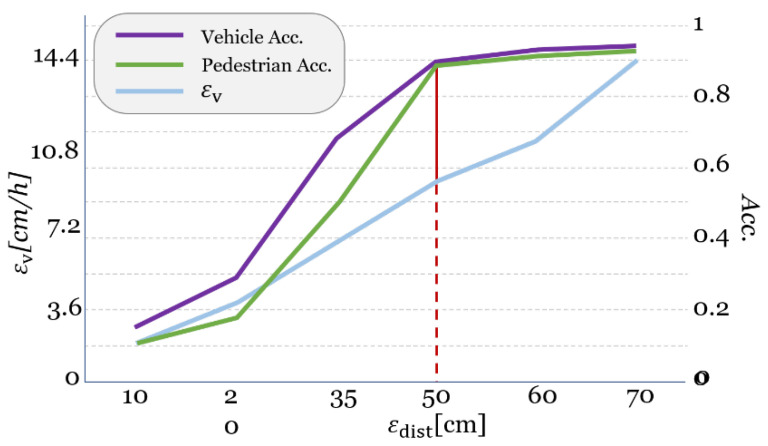
Plots for accuracies and $\varepsilon_{v}$ by $\varepsilon_{dist}$/.

For every distance tolerance, we can derive the speed tolerance. As described in Figure 12, $\varepsilon_{v}$ can be calculated with maximum potential distance tolerance between two consecutive frames, and divided by the time interval between those frames as follows:

(10) $$\varepsilon_{v}=2\times \frac{\varepsilon_{dist}}{\left(R/FPS\right)}$$

where R is the number of the skipped frames in video footage, FPS is frame-per-second, and R/FPS means time interval. As seen in Equation (10), $\varepsilon_{v}$ increases linearly in proportion to $\varepsilon_{dist}$. Thus, the optimal $\varepsilon_{dist}$ is 50 cm when considering $\varepsilon_{v}$ and accuracy, as represented in Figure 13. In our experiment, we set the time interval, R/FPS, at about 0.4 regardless of FPS. According to the above formula, the speed tolerance is about 2.5 m/s, or 9.0 km/h, when distance tolerance is 50 cm.

## 6. Analysis of Potential Collision Risky Behaviors

In this section, we analyze the potential collision risks based on the extracted behavioral features following three scenarios: (1) using distributions of vehicles’ speeds and PSMs by spots; (2) investigating driver stopping behaviors when there are pedestrians on the crosswalk; and (3) considering PSMs together with stopping behaviors.

### 6.1. Analyzing Vehicles’ Speeds and PSMs by Spots

Table 6 shows statistical values of average car speeds in each spot.

The maximum average speeds are in the range of about 51.3 to 87.5 km/h, and minimum values range from 2.2 to 9.4 km/h. The overall distributions are skewed right since many cars move slowly in these areas. The speed limit for all spots with school zones is 30 km/h. When considering that mean values in all spots are near or under the regulation speed, then these are reasonable values.

In general, cars tend to move faster when there are no pedestrians present, and slow down when there are pedestrians. We can observe these tendencies by separating the average vehicle speeds into car-only scenes and interactive scenes as seen in Table 6. In all spots, the speeds in interactive scenes are lower than those in car-only scenes. 

Spot C is the only location where the average speeds exceeded the speed limit (30 km/h). This may be related to the number of lanes and whether a speed camera is deployed. First, Spot C has four lanes, more lanes than any other spot except Spot F; generally, higher speed limits apply when there are more lanes, but the speed limit in Spot C remains 30 km/h because it is designated as a school zone. Second, Spot F matches Spot C in the number of lanes, speed limit, signalized crosswalk, and school zone designation, but Spot F has a speed camera, missing from Spot C (refer Table 1). From this example, we can hypothesize that when the number of lanes increases, vehicle speeds increase, but a speed camera can suppress such a tendency.

Next, we analyzed the extracted PSM distributions. Note that PSM counts how many seconds it takes for a car to pass through the same point after a pedestrian passes it, thus quantifying the potential risk of a vehicle–pedestrian collision. In our experiment, we filtered out the negative values and only looked at cars passing behind the pedestrians (negative PSM values mean that the car passed before the pedestrian). Then, we differentiated between the signalized crosswalks (spots A, B, C, and F) vs. unsignalized crosswalks (spots D, E, G, H, and I). 

**Figure 14:**
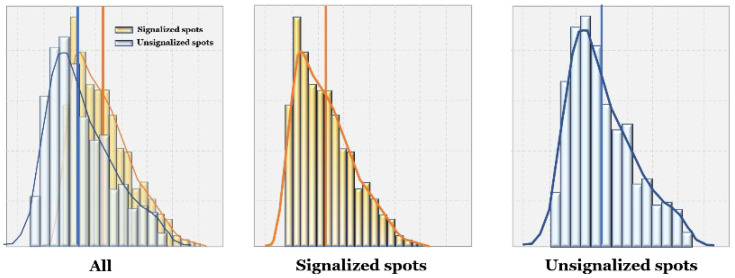
Distributions of PSMs in signalized and unsignalized spots.

Figure 14 shows the distributions of positive PSM in all signalized vs. unsignalized spots. It represents the ranges and mean values of PSM; PSMs were higher on average in signalized crosswalks than those in unsignalized crosswalks. In addition, the peak of the distribution across all signalized spots is higher, since the traffic signal forces some time to pass before cars can cross the pedestrian’s path. Without the signal, the distribution peaks are closer to zero, indicating cars are not willing to wait and give pedestrians the safety margin before passing. 

**Figure 15:**
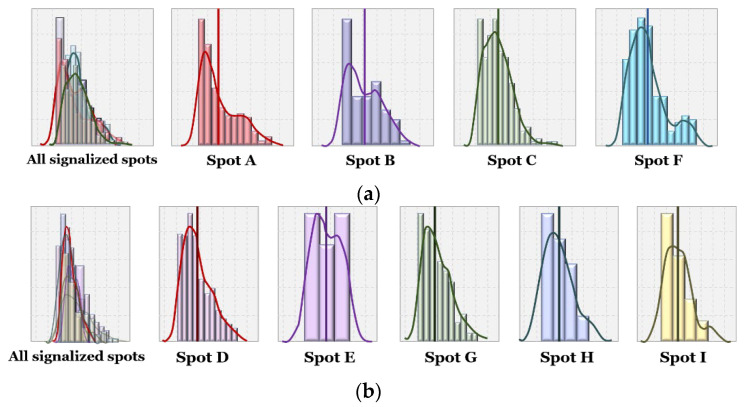
Distributions of PSMs in (**a**) signalized spots, and (**b**) unsignalized spots.

Figure 15a,b show distributions of PSM at each spot. In Figure 15a, we can observe that in signalized spots, wider roads lead to higher PSM, possibly because of longer signal cycles for pedestrian crossing. Spots C and D each have four lanes, wider than Spot A (two lanes) and B (three lanes), and their PSM distributions are further to the right. 

Meanwhile, in unsignalized crosswalks, the overall distributions are similar to each other, and we did not observe a relationship between road width and PSM distribution. Spot G stood out, with PSM distribution further right of the others; one reason could be its slower vehicle speeds overall. Since it is in a residential area, it has a particularly high floating population (especially students) during rush hour. In addition, there are road intersections close to either side of the crosswalk (see Figure 2g), forcing slower speeds and more careful maneuvering for vehicles, who in turn give pedestrians plenty of crossing time.

### 6.2. Analyzing Pedestrian’s Potential Risk near Crosswalks Based on Car Stopping Behaviors

In this sub-section, we analyzed whether or not vehicles stopped before passing the crosswalk when a pedestrian was present, and the distance they stopped from the crosswalk. Generally, vehicles may stop for a variety of reasons such as parking on the shoulder, waiting for a traffic signal, or allowing pedestrians the right-of-way. To precisely count the scenes when the driver stopped to ensure pedestrian safety, we chose 10 m as a baseline distance; if a car stopped within 10 m from the crosswalk, with a pedestrian in the crosswalk or CIA, we assumed they were reacting to the pedestrian’s presence. 

**Figure 16:**
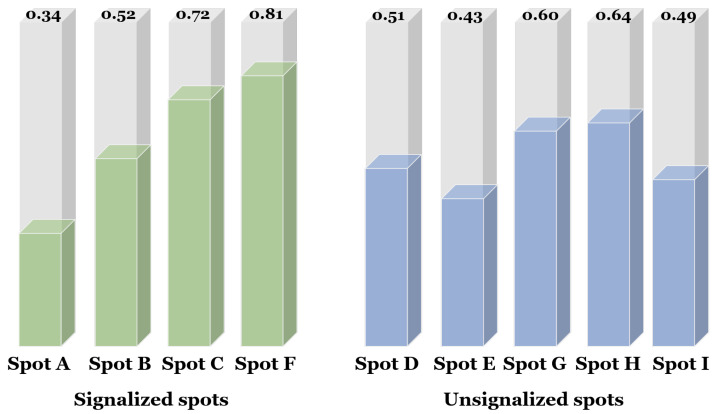
The percentages of drivers stopping within 10 m from crosswalks for scenes with pedestrians on crosswalks.

Figure 16 shows the percentages of vehicles that stopped within 10m before passing the crosswalks when pedestrians crossed the streets in signalized and unsignalized spots, respectively. First, among signalized spots, Spot A has the lowest percentage of drivers stopping. The reason could be related to the width of lanes. Spot A has just two lanes, but other signalized spots have three or more lanes. It can be interpreted that the drivers on the narrow road are reluctant to wait for the signal, so they would violate the signal. Spot F has a higher percentage than those in other spots. It can be seen that the installation of the speed camera has a deterrent force that makes the drivers obey the signal. In this experiment, we analyzed only behaviors of vehicles and pedestrians, not considering signal phases together. Note that the coexistence of the passing vehicle and crossing pedestrian implies that one of the traffic participants violates the traffic signal threatening driving safety regardless of the signal. 

Meanwhile, in unsignalized spots, especially spots G and H, most drivers did not stop before passing the crosswalk. Spot H had a relatively high stopping percentage, perhaps due to its safety features such as a red urethane pavement and “school zone” lettering on the road, as well as safety fences on both sides of the road. Spot G also had a high stopping percentage. However, since there were no signal lights, drivers were less likely to perform the required safe behavior (stopping before the crosswalk until pedestrians have cleared the area). In particular, half or more of the drivers in spots D, E, and I failed to stop when pedestrians were on the road, despite the designation of school zones. In these spots, the further proactive response seems necessary to encourage stopping for pedestrians, and prevent accidents before they occur.

### 6.3. Analyzing Car Behaviors with PSM and Car Stopping near the Unsignalized Crosswalk

In this sub-section, we analyzed driver-stopping behaviors with PSM values at unsignalized crosswalks. PSM is a simple feature that can provide implicative information for vehicle and pedestrian behaviors. Since PSM is the time difference between when a pedestrian passed a certain point and when the vehicle arrived at the same point, a positive PSM value means that the pedestrian crossed first, and a negative value means that the vehicle passed first. Since the latter implies that the vehicle failed to yield to the pedestrian in the crosswalk, negative PSM values generally present more risk than positive values. In either case, collision risk increases as PSM approaches zero. We only considered scenes in unsignalized spots in this sub-section, since yielding behavior and PSM at signalized crosswalks greatly depend on the traffic signal at the time of encounter.

In our experiment, we studied scenes occurring within various ranges of PSM, and measured the likelihood of a vehicle stopping before the crosswalk with a pedestrian present (using 10 m as a baseline distance). First, we categorized the continuous PSM values into eight groups by signs and quartiles, using a combined distribution accounting for all scenes in the five unsignalized crosswalks.

**Figure 17:**
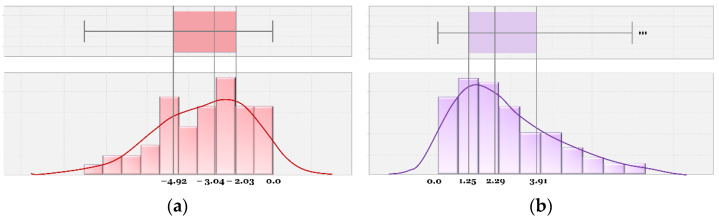
Distributions of PSMs in (**a**) signalized, and (**b**) unsignalized spots.

However, simply merging these distributions would bias the result toward the distribution of higher-traffic areas. For example, if there were 100 and 800 scenes in two regions A and B, respectively, the merged distribution across these two regions would be more affected by scenes occurring in B. Thus, we calculated the weight of each distribution relative to the whole:

(11) $$w_{i}=1-\frac{\left|D_{i}\right|}{\left|D\right|}$$

where $\left|D\right|$ is the total number of scenes in unsignalized spots (spots D, E, G, H, and I) and $\left|D_{i}\right|$ is the number of scenes in each spot. We then multiplied by $w_{i}$ to normalize the scene frequencies in spot i. As a result, Figure 17a,b represent the combined, weighted distributions of PSM values across all scenes in unsignalized crosswalks. 

From these distributions, we split between the positive and negative PSM values, and within each by quartile, to yield the following PSM ranges: (1) under −4.92; (2) −4.92 to −3.04; (3) −3.04 to −2.03; (4) −2.03 to 0; (5) 0 to 1.25; (6) 1.25 to 2.29; (7) 2.29 to 3.91; and (8) over 3.91, denoted by ranges 1 to 8, respectively. Then, we compared the stopping percentages within each PSM range. 

**Figure 18:**
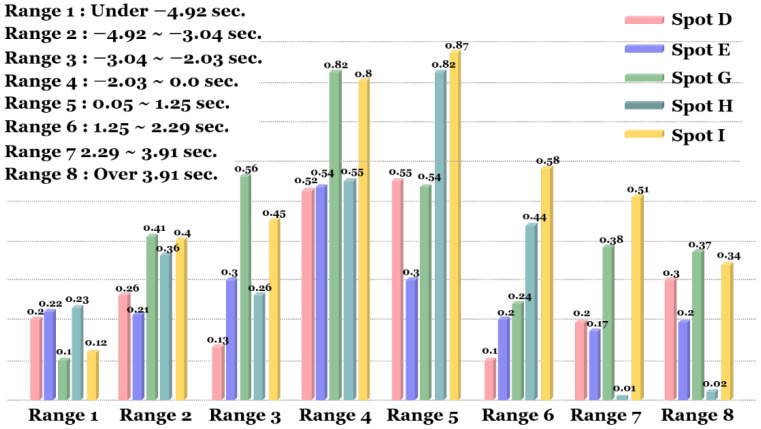
The percentages of drivers stopping before the crosswalk by PSM range.

In Figure 18, ranges 1, 2, 3, 6, 7, and 8 are relatively distant groups from zero, and ranges 4 and 5 present the greatest risk, with safety margins within 1–2 s. We can observe that as margins increase, vehicles are less likely to stop at the crosswalk.

Ideally, for small but positive PSM scenes, we would want to see the highest stopping percentages in order to minimize the risk of collision with pedestrians. However, within range 5 (PSM between 0 and 1.25 s), most cars in spot E did not stop. This could result from two possible behaviors: (1) drivers did not stop, but decelerated while passing ahead of pedestrians; or (2) drivers did not stop nor decelerate, and narrowly avoided collisions with pedestrians. Thus, Spot E represents an anomaly, since stopping percentages for other spots in these low-margin ranges are at least 50%; since it presents a greater risk of collision, we would want to understand why and proactively address the issue.

Meanwhile, we can see that at larger PSM margins, especially ranges 2, 3, 7, and 8, stopping percentages are highest in spots G and I. We hypothesize that this is because G and I have no fences separating the road from the sidewalk, unlike the other unsignalized spots. Without the fences, drivers may be forced to drive more cautiously through the area, since pedestrians could potentially enter the road at any point along with the approach to the crosswalk. In these areas, adding safety features such as sidewalk fences could negatively affect the behavior of vehicles and pedestrians, by removing the uncertainty that forces driver caution and more frequent stopping.

### 6.4. Discussions

The proposed approaches in this research had three main objectives: (1) to process the video data as one sequence from the entire video footage; (2) to automatically extract objects’ behaviors affecting the likelihood of potentially dangerous situations between vehicles and pedestrians; and (3) to analyze behavioral features and relationships among them by camera locations. Unlike our previous study [29], this research analyzed a variety of potential collision risky behaviors, and expanded the scale to more cameras over longer time frames by capturing diverse road environments, such as signalized and unsignalized crosswalks. This study is an extension of our previous work [21], being similar to the object detection and tracking parts. However, this study handled more video data from multiple spots, unlike the previous one, and further aimed at extracting behavioral features including risky behavioral characteristics such as PSM as well as simple features such as speed and position. Furthermore, this study focused on analyzing these features in terms of potential risks between vehicle and pedestrian, unlike our previous one [21].

In our experiments, we extracted time- and distance-based various behavioral features affecting potential risks such as vehicle’s speed, pedestrian’s speed, vehicle’s acceleration, and PSM. In order to observe how sensitive drivers were to the risk of pedestrian collision, we categorized scenes as car-only vs. vehicle–pedestrian interactive scenes. Then, we performed three analyses: (1) distributions of the average car speeds and PSMs by spots; (2) percentages of vehicles stopping when pedestrians are present in or near the crosswalk; and (3) stopping behaviors relative to PSM. We observed how vehicle speeds responded to road environments, and how they changed when approaching pedestrians.

One limitation of this system is the lack of an interface to perform a comprehensive analysis of various situations. For example, the size and complexity of the generated dataset make it difficult to answer questions such as: “at unsignalized crosswalks, when the average vehicle speed is between 30 and 40 km/h, between 8 am and 9 am, and pedestrians are present, what is the acceleration state of the vehicles in each spot?” or “when PSM is in range of −1 to 0, what were vehicle speeds in school zones in the evening?” In order to address these challenges, we need to classify the given behavioral features according to their characteristics to enable multidimensional analysis, such as an online analytical process (OLAP) and data mining techniques. This would allow administrators (e.g., transportation engineers or city planners) to interpret the behavior features, understand existing areas, design alternative roads/crosswalks/intersections, and test the impact of these physical changes.

## 7. Conclusions

In this study, we proposed a new approach to obtain the potential risky behaviors of vehicles and pedestrians from CCTV cameras deployed on the roads. The keys are: (1) to process the video data as one sequence from motioned-scene partitioning to object tracking; (2) to extract automatically the behavioral features of vehicles and pedestrians affecting the likelihood of potential collision risks between them; and (3) to analyze behavioral features and relationships among them by camera locations. We validated the feasibility of the proposed analysis system by applying it to actual crosswalks in Osan City, Republic of Korea.

This study was motivated by a lack of a vision-based analysis approach for road users’ risky behaviors, by automatically using video processing and deep learning-based techniques. These analyses can provide powerful and useful information for decision makers to improve and make road environments safer. However, our approaches themselves would not identify the best control or traffic calming measures to prevent traffic accidents. We hypothesize that it can provide practitioners with enough clues to support further investigation through other means. Furthermore, traffic safety administrators and/or policy makers must collaborate using these clues to improve the safety of the spaces. Our goal in developing this system was to aid in this collaboration, by making it faster, cheaper, and easier to collect objective information about the behavior of drivers at places where pedestrians face the greatest risks.

## Figures and Tables

**Table 1:** Information of the obtained spots.

Spot Code	Cam. Name	Crosswalk Length (m)	School Zone	Speed Cam.	The Number of Lanes	Signal Light	Speed Limit (km/h)	Frame Size	Frame-per-Sec (FPS)
A	Unam Elementary school, back gate #2	about 8 m	+	×	2	×	30 km/h	1920 × 1080	25
B	Yangsan Elementary school, main gate #1	about 11 m	+	×	3	×	30 km/h	1920 × 1080	25
C	Gohyeon Elementary school, back gate #2	about 20 m	+	×	4	×	30 km/h	1920 × 1080	25
D	Municipal Southern Welfare/Daycare center #3	about 7 m	+	×	2	+	30 km/h	1280 × 720	30
E	iFun daycare center #2	about 8 m	+	×	2	+	30 km/h	1280 × 720	30
F	Daeho Elementary school opposite side #3	about 23 m	+	+	4	×	30 km/h	1280 × 720	30
G	Segyo complex #9 back gate #2	about 8 m	×	×	2	+	30 km/h	1280 × 720	15
H	iNoritor daycare center #2	about 8 m	+	×	2	+	30 km/h	1280 × 720	11
I	Kids-mom daycare center #3	about 7 m	+	×	2	+	30 km/h	1920 × 1080	25

**Note:** +: Yes ×: No.

**Table 2:** The number of the extracted scenes after preprocessing.

Spot Code	The Number of Scenes (After Preprocessing)		The Number of Total Frames	Avg. Frames in One Scene (Ranges)
	Car-Only Scenes	Interactive Scenes		
A	4221		136,189	32.26 frames (1.29 s)
	2681	1540		
B	2908		86,249	29.66 frames (1.18 s)
	1721	1187		
C	4111		382,980	93.16 frames (3.72 s)
	2321	1790		
D	6955		219,240	31.52 frames (1.05 s)
	4633	2322		
E	3876		125,935	32.49 frames (1.08 s)
	2481	1395		
F	7587		377,752	44.51 frames (1.48 s)
	6494	1093		
G	5612		175,247	31.22 frames (2.08 s)
	3533	2079		
H	2845		47,468	16.68 frames (1.11 s)
	1843	1002		
I	7775		260,260	33.47 frames (1.34 s)
	4572	3203		

**Table 3:** The extracted features in our experiment.

Target Object	Feature Name	Description	Example
Vehicle	Speed	Vehicle speeds change by frames Unit: km/h	[14.3, 12.0, 9.8, 4.3, 7.8, 12.1…]
	Position	Vehicle positions change based on a crosswalk by frames Represented as “before crosswalk”, “on crosswalk” or “after crosswalk”	[before crosswalk, on crosswalk] [before crosswalk, on crosswalk, after crosswalk]
	Acceleration	Vehicle accelerations change by frames Represented as “acceleration (acc)”, “deceleration (dec)” or “no change (nc)”	[acc, nc] [nc] [acc, nc, acc]
	Crosswalk distance	Distance changes between vehicles and crosswalks by frame Unit: m	[4.1, 3.3, 1.9, …]
	Car stops before crosswalk	Whether the vehicles stopped before passing the crosswalk in one scene Represented as “stop” or “no stop”	stop no stop
Pedestrian	Speed	Pedestrian speeds change by frame Unit: km/h	[2.3, 2.0, 1.9, …]
	Position	Pedestrian positions change by frames Represented as “sidewalk”, “crosswalk” or “CIA (crosswalk-influenced area)”	[sidewalk, CIA, sidewalk] [crosswalk]
Vehicle–pedestrian interaction	Distance	Distance changes between vehicle and pedestrian by frame Unit: m	[4.1, 3.3, 1.9, …]
	Relative position	Relative positions list between vehicle and pedestrian by frame “Front” means pedestrian is on the front side of the car, and “Behind” means the pedestrian is on the back side of the car	[Front, Front, Front, Behind, Behind] [Behind, Behind, Front]
	Pedestrian safety margin	Pedestrian safety margin in one scene Unit: sec.	3.2 −1.5

**Table 4:** Results of trajectory validation based on three criteria.

Result of Trajectory without Kalman Filter (Car Threshold = 100, Pedestrian Threshold = 50)					
Spot Code	# of Scenes	The Number of Error Frames			
		Connectivity	Crossing	Directivity	Accuracy
Spot A	4789	45	98	305	0.91
Spot B	3195	35	75	285	0.88
Spot C	5311	32	112	401	0.90
Spot D	7304	49	155	491	0.90
Spot E	4261	54	98	358	0.88
Spot F	8036	61	187	652	0.89
Spot G	6259	55	138	499	0.89
Spot H	3295	25	59	441	0.84
Spot I	7940	35	90	595	0.91
**Average**		**291**	**1012**	**4027**	**0.89**
**Result of trajectory without Kalman filter** **(Car threshold = 100, pedestrian threshold = 50)**					
**Spot code**	**# of scenes**	**The number of error frames**			
		**Connectivity**	**Crossing**	**Directivity**	**Accuracy**
Spot A	4789	25	66	194	0.94
Spot B	3195	21	58	201	0.91
Spot C	5311	22	74	298	0.93
Spot D	7304	40	101	347	0.93
Spot E	4261	41	59	256	0.91
Spot F	8036	45	111	515	0.92
Spot G	6259	35	77	398	0.92
Spot H	3295	14	32	387	0.86
Spot I	7940	28	47	457	0.93
**Average**		**271**	**635**	**3053**	**0.92**

**Table 5:** Results of accuracy using tolerance for vehicle and pedestrian in each spot.

Spot Code	Tolerance (cm)											
	Target Object											
	10		20		35		50		60		70	
	V	P	V	P	V	P	V	P	V	P	V	P
A	0.18	0.10	0.36	0.23	0.69	0.51	0.93	0.89	0.95	0.90	0.95	0.91
B	0.17	0.09	0.31	0.23	0.70	0.48	0.88	0.87	0.97	0.88	0.98	0.97
C	0.10	0.10	0.24	0.19	0.64	0.52	0.90	0.90	0.95	0.87	0.96	0.88
D	0.25	0.11	0.32	0.14	0.72	0.53	0.90	0.90	0.95	0.91	0.97	0.91
E	0.17	0.14	0.28	0.11	0.71	0.49	0.89	0.87	0.96	0.95	0.97	0.95
F	0.12	0.12	0.29	0.17	0.69	0.56	0.90	0.93	0.94	0.90	0.96	0.94
G	0.17	0.12	0.37	0.21	0.72	0.51	0.89	0.91	0.90	0.94	0.92	0.93
H	0.14	0.13	0.25	0.20	0.70	0.46	0.90	0.91	0.92	0.92	0.93	0.92
I	0.11	0.10	0.23	0.17	0.68	0.45	0.89	0.84	0.94	0.92	0.96	0.94
**Average**	0.16	0.11	0.30	0.18	0.69	0.50	**0.90**	**0.89**	0.94	0.91	0.95	0.93

**Note**. V: Vehicle, P: Pedestrian.

**Table 6:** Average vehicle speed information in all spots by scene types.

Spot Code	All Scenes			Types of Scenes	
	Max. (km/h)	Min. (km/h)	Mean (km/h)	Avg. of Car-Only Scene (km/h)	Avg. of Interactive Scene (km/h)
A	71.3	3.6	18.2	20.5	12.2
B	87.5	4.4	24.5	25.9	16.2
C	75.4	6.5	36.5	41.7	21.7
D	79.7	4.1	18.1	18.4	14.6
E	68.1	2.2	22.3	22.3	17.6
F	51.3	3.9	20.9	21.2	11.3
G	63.9	9.4	14.0	14.2	9.4
H	59.2	3.3	21.4	21.5	14.7
I	70.2	7.4	33.8	34.	19.8
